# A cross-sectional study evaluating health-related quality of life of Chinese pediatric patients with hematological malignancies using EQ-5D-Y

**DOI:** 10.3389/fpubh.2022.1050835

**Published:** 2023-01-11

**Authors:** Yue Sun, Hui-Jun Zhou, Anle Shen, Bin Wu, Wei Wang, Nan Luo, Pei Wang

**Affiliations:** ^1^Division of Medical Affairs, Ruijin Hospital, Shanghai Jiaotong University School of Medicine, Shanghai, China; ^2^Key Lab of Health Technology Assessment, National Health Commission of the People's Republic of China (Fudan University), Shanghai, China; ^3^Business School, University of Shanghai for Science and Technology, Shanghai, China; ^4^Department of Pharmacy, Shanghai Children's Medical Centre, School of Medicine, Shanghai Jiaotong University, Shanghai, China; ^5^Medical Decision and Economic Group, Department of Pharmacy, Renji Hospital, School of Medicine, Shanghai Jiaotong University, Shanghai, China; ^6^School of Public Health, Fudan University, Shanghai, China; ^7^Saw Swee Hock School of Public Health, National University of Singapore, Singapore, Singapore

**Keywords:** health-related quality of life, health utility assessment, pediatric patient, hematological malignancies, EQ-5D-Y

## Abstract

**Background:**

The study aimed to assess health-related quality of life (HRQoL) and to estimate the health utility of pediatric patients with hematological malignancies (HMs) in China.

**Method:**

A cross-sectional study recruited a series of pediatric inpatients diagnosed with HM from November 2018 to May 2019 in the Shanghai Children's Medical Center. Subjects were interviewed to collect sociodemographic information about themselves and their guardians. The EQ-5D-Y was completed by each patient to rate their own HRQoL, which later derived the health utility. The health status was also assessed by clinicians following the Eastern Cooperative Oncology Group (ECOG) system. Upon the descriptive analysis and univariate analysis, multivariate generalized linear models were built to explore the associations of risk factors with HRQoL measures of utility, Visual Analog Scale (VAS) score, and the five EQ-5D-Y domains.

**Results:**

The 96 subjects had a mean age of 10.5 years and included 62 (64.4%) boys. There were 46 (47.9%) and 25 (26.0%) children diagnosed with acute lymphoblastic leukemia and non-Hodgkin's lymphoma, respectively. The means (SD) of utility and EQ-VAS scores were 0.88 (0.10) and 85.8 (15.1), respectively. Twenty-six (27.1%) patients were graded poor health by the ECOG standard (score 2/3). Both univariate and multivariate analyses found strong correlations between ECOG and HRQoL. After adjusting for covariates, poor ECOG score was significantly associated with an impaired utility and VAS of −0.103 and −8.65, respectively. With regard to individual HRQoL domains, worse ECOG was more likely to report health problems with an increased risk of 2.94 to 12.50; residence, income, guardians' education, and disease duration were also found to be significantly related to either the utility or certain health domains.

**Conclusion:**

The HRQoL of Chinese pediatric patients with HM is considered relatively poor and of great concern to healthcare. With the strong correlations between EQ-5D-Y-related HRQoL measures and the traditional clinical index ECOG, the EQ-5D-Y is able to provide valuable evidence for clinical decision-making at the individual level. At the same time, its health utility can inform resource allocation at a macro level.

## Introduction

Health-related quality of life (HRQoL) of pediatric patients is an important topic in resource allocation and healthcare service optimization in pediatric cancer care. Health policies informed by utility-based economic evaluations require reliable estimates of utility (1). To yield valid HRQoL for pediatric patients, an age-appropriate instrument was required given that HRQoL measurement in children is different from adults. The EQ-5D-Y (youth) is a generic HRQoL instrument adapted from the adult EQ-5D questionnaire, specifically to measure the health status of children between 8 and 18 years (2). As one of the EQ-5D questionnaire series, it is able to derive health utility from the five dimensions describing health status. Its psychometric properties have been validated in local pediatric populations (3), laying the foundation to be applied in clinical practice.

Hematological malignancies (HMs) are common tumors in the child populations and made the second highest mortality among pediatric tumors in China (4). The prevalence of HMs was 4.2/100,000 in children younger than 14 years old, and ~15,000 new cases were diagnosed every year in those below 18 years (5). The treatment for HM was costly with annual treatment costs between 100 and 300 K Chinese Yuan (CNY) for one patient, thus imposing a heavy financial burden on the family and society as well (6).

The health of patients with HM was debilitated not only by the HM-specific symptoms such as anemia, headache, fatigue, and pain but also by the adverse effects related to radiotherapy and chemotherapy. Patients had to be hospitalized frequently in the course of treatment and recovery (7, 8). Studies have reported worse health status of adult patients with HM compared to the general population (9). The situation can only be worse in the younger patients who are usually unable to handle the health problems on their own. After all, children are in development when their cognitive, physiological, and psychological functions are not mature. Both overseas and local studies in China have reported decreased HRQoL in pediatric patients with HM (10–12). Their physical activities, school performance, and HRQoL were impaired and associated with younger age, inattentive parenting patterns, lower guardians' educational level, and poor economic status of the family (8–10, 12).

With the advancement of HM care, children with HM now can survive to adulthood and most of them expect a normal life span. Therefore, the HRQoL of such patients turned up to be a practical issue. Unfortunately, the evidence of HRQoL in this population is scarce so far in China. Health utility was hardly reported. The cost-utility analyses hence lack the primary input of utility to support evidence-based policymaking in the care of children with HMs.

Under this circumstance, we hypothesized that Chinese pediatric patients with HM have impaired HRQoL and lower utility compared to their peers. The utility data about this population would accelerate the economic studies to better the oncological care of HM. Therefore, we conducted this study: (1) to assess the HRQoL in children/adolescents who were diagnosed with HM in China; (2) to estimate their health utility; and (3) to determine the significant factors for the HRQoL.

## Methods

### Study sample

The study population was defined as Chinese pediatric patients diagnosed with any type of HM in mainland China. Due to the specificity of the disease, we chose Shanghai Children's Medical Center, a tertiary hospital specializing in pediatric care, as the study site. A series of patients were approached once they were admitted to the hospital and settled down in the ward. The inclusion criteria were: (1) a confirmed diagnosis of HM; (2) aged 8–18 years; (3) no concurrent debilitating conditions; (4) no major invasive procedures in the past 3 months; and (5) able to understand questions. Considering the availability of eligible patients, constraints of research resources and the number of independent variables to be explored in the multivariate analysis, n = 100 was determined as the targeted sample size. During the study period from November 2018 to May 2019, the 96 eligible patients who were approached all consented to participate in the study. The informed consent form was signed by parents or next-of-kin before the patient interview and data collection.

The study adhered to the tenets of the Declaration of Helsinki involving human participants and ethics approval was obtained from the Institutional Review Board of the School of Public Health, Fudan University.

### Data collection

Data were collected through face-to-face interviews conducted by the trained interviewers with patients, their guardians, and doctors-in-charge. During the interview, information about patients' demographics, socioeconomic status, and guardians was gathered. Data about guardians including age, education level, occupation, and monthly income of the family were collected. At the same time, patients rated their own health status by completing the EQ-5D-Y questionnaire. Doctors-in-charge evaluated each patient in terms of the Eastern Cooperative Oncology Group (ECOG) performance score, mental consciousness, reactions, complexion, and petechia.

### ECOG performance score

The Eastern Cooperative Oncology Group (ECOG) performance score, which was given by the doctor-in-charge, ranks a cancer patient's ability to perform daily activities in five grades (13): 0–fully active and no performance restrictions; (1) strenuous physical activity restricted, fully ambulatory and able to carry out light work; (2) capable of self-caring but unable to work, over 50% of daytime to get up and have activities; (3) capable of only limited self-caring, confined to bed or chair over 50% of daytime; (4) completely disabled and cannot self-care, totally confined to bed or chair; (5) death. An ECOG score of 0 or 1 indicates good health while higher scores (2–4) indicate poor health (14).

### Health-related quality of life measurement

With the guidance of the trained interviewers, the patients completed the EQ-5D-Y rating of their health on the day of the survey. EQ-5D-Y comprises two sections, a descriptive system and a visual analog scale (EQ-VAS). The former asks a subject to evaluate his/her HRQoL on five dimensions, that is, “walking about,” “looking after myself,” “doing usual activities,” “having pain or discomfort,” and “feeling worried, sad, or unhappy” with each dimension categorized into three severity levels (no problems, some problems, and a lot of problems). The EQ-VAS ranging from 0 (the worst imaginable health) to 100 (the best imaginable health) inquires about the overall health rated by a patient itself. Recent HRQoL studies on Chinese pediatric patients with HM reported good or very good test–retest reliability for EQ-5D-Y as the Gwet agreement coefficients varied from 0.628 to 0.901 for the individual dimensions and intraclass correlation coefficients were 0.833 for the EQ-VAS (15). Convergent validity and known-groups validity were tested moderately in healthy Chinese children aged 8–18 years (16).

Health utility would be derived by incorporating patients' responses to the five dimensions following specific value sets for utility calculation. In this study, we were obliged to adopt the Japanese value set as the Chinese one was under development (17). As per the Japanese value set, the worst HRQoL corresponds to a utility of 0.067, while full health takes a utility of 1.0.

### Statistical analysis

Descriptive statistics summarized the demographic, socioeconomic, and clinical characteristics of the sample. The internal consistency of EQ-5D-Y in measuring HRQoL was checked with Cronbach's Alpha. Then, the HRQoL was illustrated by the proportions of “reporting problems” on the five individual dimensions. The overall HRQoL was estimated as the means (standard deviation, SD) of utility and EQ-VAS scores. The general health grades in the ECOG system were also reported as proportions. The chi-square test, *t*-test, and analysis of variance were used to compare health outcomes among different levels of each variable. Correlation analyses explored the relationship between continuous variables and utility and VAS.

Multivariate generalized linear models (GLMs) were built separately on the utility and EQ-VAS score to explore their associations with potential risk factors, that is, patients' age, gender, education level (middle school vs. primary school), residence (rural area vs. urban), disease duration, diagnosis, and ECOG score (poor vs. good), and their guardians' education level (primary school or below/middle school vs. college or above), and monthly income (< CNY 5,000 vs. ≥5,000) (18, 19). For the categorical variable put in the model, each category must have 20 observations at a minimum. To assess the relationship between individual health dimensions and the factors, five GLMs were established with a binary dependent variable indicating the existence of any problems in each of the five dimensions.

The analyses were performed using SPSS (version 19) at a significance level of 0.05.

## Results

As summarized in Table 1, the patient's mean age (SD) was 10.5 (2.2) years, with the boys accounting for 64.6%. The majority (79.2%) of the sample were in primary education. The most common diagnosis was acute lymphoblastic leukemia (47.9%), followed by non-Hodgkin's lymphoma (26.0%). The mean (SD) disease duration was 8.3 (16.5) months with a wide range from 3 days to 7.6 years. There were 16 (16.7%) and 54 (56.3%) patients graded at ECOG score of 0 or 1 respectively, indicating good physical function.

**Table 1:** Demographic, socioeconomic, and clinical characteristics of patients with hematological malignancies.

**Patients (%**, ***N*****)**		**Guardians (%**, ***N*****)**	
Age (year)^*^	10.5 (2.2)	Age (year)^*^	40.1 (9.3)
Gender		Marriage	
Boy	64.6% (62)	Unmarried	3.1% (3)
Girl	35.4% (34)	Married	95.8% (92)
Education		Religion	
Primary school	79.2% (76)	Yes	16.7% (16)
Middle school	20.8% (20)	No	78.1% (75)
Residence		Relationship	
Urban	53.1% (51)	Father	20.8% (20)
Rural area	44.8% (43)	Mother	67.7% (65)
Diagnosis		Other	11.5% (11)
Acute lymphoblastic leukemia	47.9% (46)	Occupation	
Non-Hodgkin's lymphoma	26.0% (25)	Employee	21.9% (21)
Acute myeloid leukemia	10.4% (10)	Civil servant/public institution	12.5% (12)
Rhabdomyosarcoma	4.2% (4)	Housewife	19.8% (19)
Osteosarcoma	2.1% (2)	Retired	3.1% (3)
Other hematological malignancies	9.4% (9)	Peasant	18.8% (18)
Disease duration (months)^*^	8.3 (16.5)	Temporary worker	3.1% (3)
ECOG		Unemployed	15.6% (15)
0	16.7% (16)	Education	
1	56.3% (54)	Primary school or below	18.8% (18)
2	20.8% (20)	Middle school	45.8% (44)
3	6.3% (6)	College or above	33.3% (32)
Clinical characteristics		Monthly income (CNY)	
Mental consciousness (Good)	100.0% (96)	< 5,000	40.6% (39)
Reactions (Good)	100.0% (96)	5,000–10,000	16.7% (16)
Complexion (Good)	91.7% (88)	10,001–30,000	12.5% (12)
Petechia (No)	86.5% (83)	>30,000	6.3% (6)

* Mean (SD).

**Figure 1:**
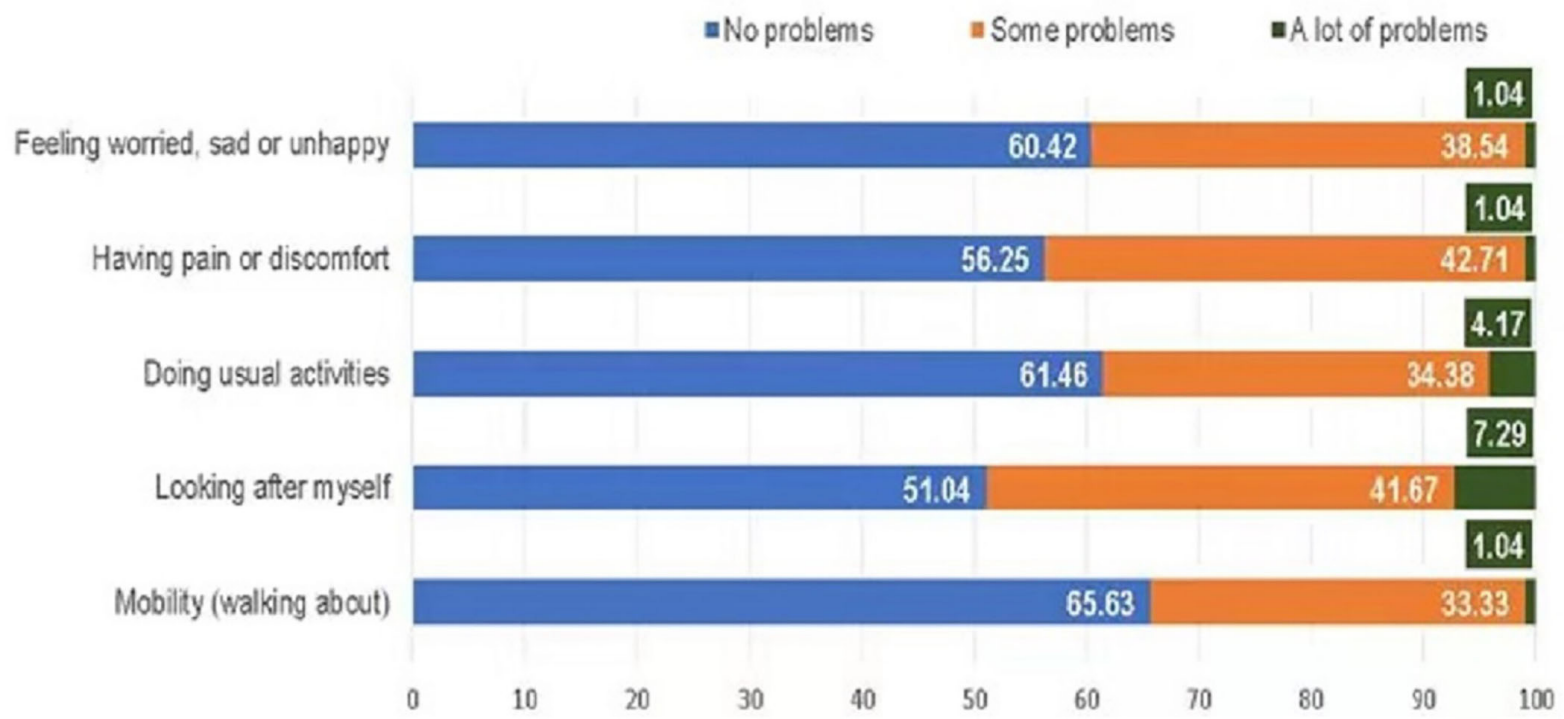
Health-related quality of life profile in EQ-5D-Y dimensions. Numbers are percentages.

Cronbach's alpha of EQ-5D-Y was 0.713, meaning the internal consistency of EQ-5D-Y in measuring this sample was acceptable. The utility score had a mean (SD) of 0.88 (0.10) and a range from 0.508 to 1. The self-rated global health by VAS score ranged from 45 to 100 around the mean (SD) of 85.8 (15.1). The HRQoL profile composed of the individual health domains is shown in Figure 1. In general, about 51.0–65.6% of patients had no problems on the five dimensions, and patients who had some problems accounted for 33.3–42.7% (“mobility”: 33.3%, “having pain or discomfort”: 42.7%). The most affected domain was “looking after myself” seeing that seven (7.29%) subjects reported the worst state.

**Figure 2:**
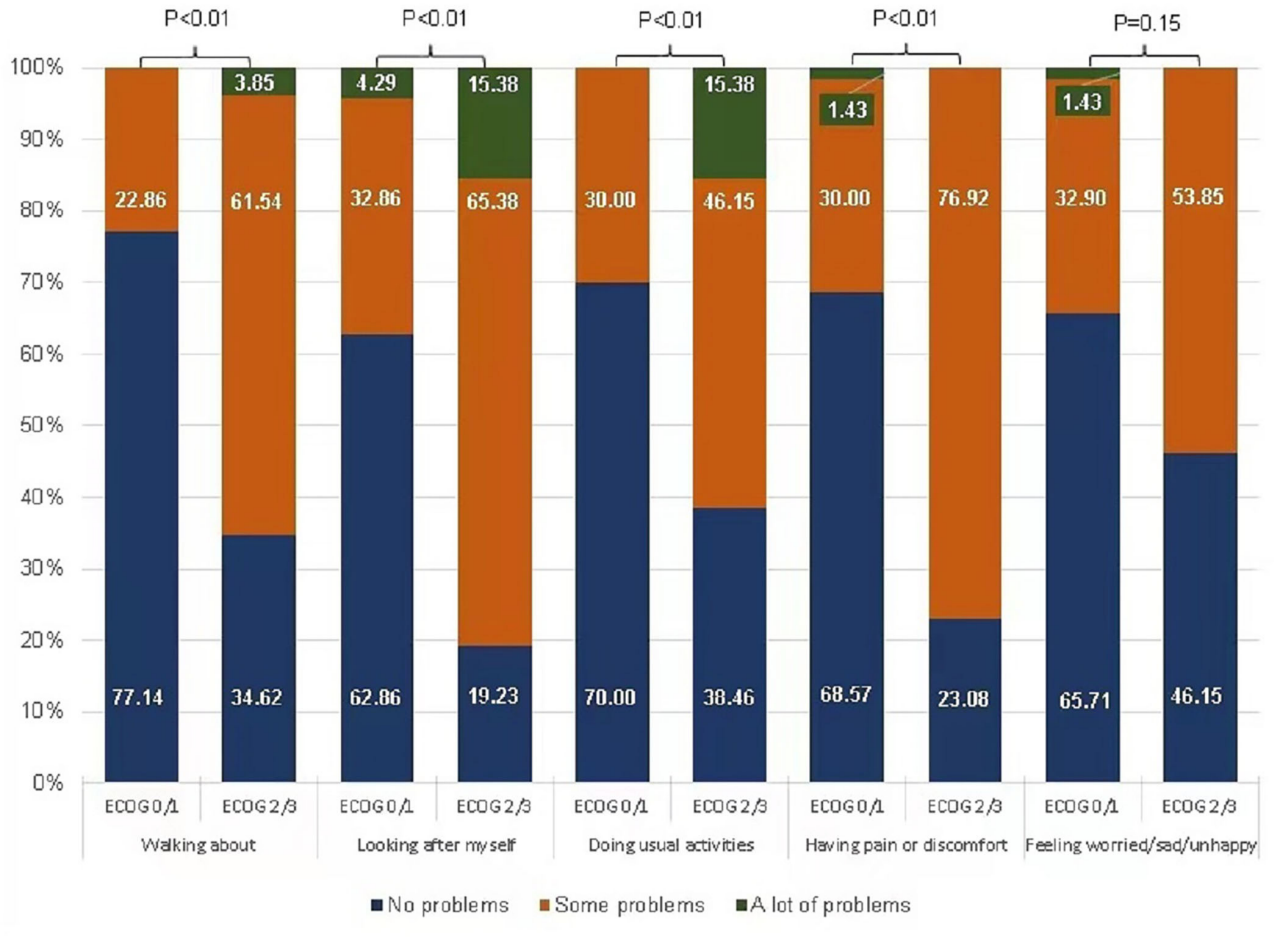
Health-related quality of life profile in EQ-5D-Y dimensions by ECOG status. Numbers are percentages.

As shown in Table 2, VAS and utility were different in categories defined by individual patient characteristics. Patients in the guard of parents finishing middle school or above rated higher VAS than those cared for by parents with an education level of primary school or below. Patients graded as having a good performance by ECOG (0/1) had mean (SD) utility and VAS of 0.91 (0.09) and 88.9 (10.7), respectively, which was significantly higher than patients graded ECOG 2/3 with the mean (SD) utility and VAS of 0.81 (0.09) and 77.3 (21.2), respectively. ECOG was linked to the HRQoL profile (Figure 2). The patients graded ECOG 2/3 reported significantly more problems in four of the five EQ-5D-Y dimensions than those graded ECOG 0/1, except for the dimension “feeling worried, sad, or unhappy.” The most affected domains in this group were “looking after myself” and “doing usual activities” both captured 15.38% of the sample reporting “a lot of problems.”

**Table 2:** Univariate analyses showing VAS and utility distribution for different risk factors.

	***N***	**Utility**		**VAS**	
		***R***	***p***	***r***	***p***
Age	96	−0.021	0.842	−0.072	0.488
Disease duration (months)	96	0.136	0.187	0.03	0.774
		Mean (SD)	*p*	Mean (SD)	*p*
**Gender**					
Female	34	0.885 (0.106)		84.21 (19.15)	
Male	62	0.877 (0.097)	0.816	86.65 (12.52)	0.453
**Education**					
Primary school	76	0.879 (0.102)		85.68 (15.73)	
Middle school	20	0.885 (0.093)	0.816	86.15 (13.06)	0.903
**Residence**					
Rural	43	0.892 (0.101)		84.44 (18.43)	
Urban	53	0.87 (0.1)	0.287	86.87 (11.92)	0.438
**Guardians' education**					
Primary school or below	20	0.855 (0.109)		76.2 (23.56)	
Middle school	44	0.897 (0.1)		89.36 (11.04)	
College or above	32	0.872 (0.094)	0.258	86.84 (10.74)	0.004
**Monthly income (CNY)**					
< 5,000	47	0.887 (0.102)		88.85 (10.3)	
≥5,000	49	0.874 (0.099)	0.534	82.84 (18.29)	0.049
**Diagnosis**					
Acute lymphoblastic leukemia	46	0.877 (0.101)		87.13 (12.62)	
Non-Hodgkin's lymphoma	25	0.902 (0.08)		89.32 (7.97)	
Others	25	0.863 (0.116)	0.389	79.76 (22.17)	0.057
**ECOG**					
Good (0/1)	70	0.908 (0.089)		88.94 (10.74)	
Poor (2, 3)	26	0.806 (0.091)	< 0.001	77.27 (21.21)	0.012

According to the GLM examining the associations of patients' characteristics with HRQoL indices (Table 3), ECOG emerged as the strongest factor to predict the concurrent utility and VAS. After adjusting for other factors, good performance by ECOG contributed to 0.103 utility and 8.65 VAS compared to poor ECOG performance. In addition, disease duration showed a significant positive association with utility with an efficiency of 0.001. Patients diagnosed with acute lymphoblastic leukemia or non-Hodgkin's lymphoma tended to achieve higher utility and VAS than those diagnosed with other types of HM; however, the differences did not demonstrate statistical significance.

**Table 3:** Multivariate analyses evaluating associations of factors with utility and VAS.

**EQ-5D-Y utility score**			**EQ-VAS score**	
	β **(95% CI)**	***P***	β **(95% CI)**	***P***
Age (years)	−0.002 (−0.014, 0.01)	0.755	−0.67 (−2.08, 0.74)	0.350
Gender (boy vs. girl)	−0.009 (−0.046, 0.027)	0.613	−1.59 (−6.34, 3.16)	0.512
Education (primary school vs. middle school)	−0.029 (−0.087, 0.029)	0.327	−3.85 (−10.99, 3.29)	0.290
Residence (rural vs. urban)	0.026 (−0.013, 0.066)	0.194	2.10 (−3.88, 8.09)	0.491
**Monthly income (CNY)**				
< 5,000 vs. ≥5,000	0.03 (−0.003, 0.064)	0.075	5.07 (−0.17, 10.3)	0.058
**Guardians' education**				
Primary school or below vs. college or above	−0.016 (−0.08, 0.048)	0.625	−3.48 (−12.73, 5.77)	0.461
Middle school vs. college or above	0.026 (−0.01, 0.061)	0.156	4.05 (−1.66, 9.75)	0.165
**Diagnosis**				
Acute lymphoblastic leukemia vs. others	0.026 (−0.022, 0.074)	0.284	4.84 (−0.62, 10.29)	0.082
Non-Hodgkin's lymphoma vs. others	0.030 (−0.021, 0.081)	0.249	4.71 (−1.43, 10.84)	0.133
Disease duration (months)	0.001 (0, 0.002)	0.014	0.060 (−0.07, 0.19)	0.344
ECOG (poor vs. good)	−0.103 (−0.14, −0.066)	< 0.001	−8.65 (−13.66, −3.64)	0.001

The odds ratio (OR) quantifying the associations of risk factors with health problems on individual EQ-5D-Y dimensions is summarized in Table 4. After controlling for the covariates, significant associations were observed between ECOG and health dimensions each, showing that poor ECOG indicated bigger probabilities of having HRQoL problems (“working about” [OR = 5.88; 95% CI: 2.08, 20.03], “looking after myself” [OR = 9.09; 95% CI: 2.56, 50.04], “doing usual activities” [OR = 3.70; 95%CI: 1.35, 11.01], “having pain or discomfort” [OR = 12.50; 95% CI: 3.85, 47.62], and “feeling worried, sad, or unhappy” [OR = 2.94; 95% CI: 1.11, 9.09]). The patients living on a monthly income below CNY 5,000 were less likely to report problems on “having pain or discomfort” (OR = 0.22; 95%CI: 0.05, 0.82) compared to those who lived on CNY 5,000 or more. Significant associations were observed between guardians' educational level with “looking after myself” and “feeling worried, sad, or unhappy” dimensions. Compared with the patients raised by parents with college or higher education, patients whose guardians' educational level was middle school tended to report fewer problems (OR = 0.28; 95% CI: 0.08, 0.92) on “feeling worried, sad, or unhappy,” while those raised by parents with the educational level of primary school or below reported more problems on “looking after myself” with an increased likelihood (OR = 10.05; 95% CI: 1.35, 92.73). In addition, patients in rural areas significantly reported fewer problems in “looking after myself” dimension compared to those living in the urban area (OR = 0.07; 95% CI: 0.01, 0.28).

**Table 4:** Multivariate analyses evaluating associations of factors with each of the five health domains.

**Mobility**			**Looking after myself**		**Doing usual activities**		**Having pain or discomfort**		**Feeling worried, sad or unhappy**	
	**OR (95% CI)**	***P***	**OR (95% CI)**	***P***	**OR (95% CI)**	***P***	**OR (95% CI)**	***P***	**OR (95% CI)**	***P***
Age (years)	0.97 (0.69, 1.37)	0.867	0.92 (0.63, 1.32)	0.631	0.87 (0.61, 1.22)	0.383	1.02 (0.71, 1.45)	0.919	1.07 (0.78, 1.46)	0.686
Gender (girl vs. boy)	1.79 (0.62, 5.31)	0.246	0.66 (0.23, 1.92)	0.462	1.64 (0.6, 4.63)	0.317	2.19 (0.76, 6.72)	0.155	0.85 (0.31, 2.29)	0.743
Education (middle school vs. primary school)	1.72 (0.32, 9.82)	0.564	1.08 (0.17, 7.09)	0.933	0.60 (0.11, 3.13)	0.509	1.35 (0.23, 8.36)	0.720	1.91 (0.4, 9.73)	0.413
Residence (rural area vs. urban)	0.63 (0.18, 2.13)	0.449	0.07 (0.01, 0.28)	< 0.001	0.27 (0.07, 0.87)	0.033	0.70 (0.19, 2.48)	0.567	1.67 (0.53, 5.54)	0.397
**Monthly income (CNY)**										
< 5,000 vs. ≥ 5,000	0.86 (0.25, 2.89)	0.797	0.33 (0.07, 1.25)	0.080	1.33 (0.42, 4.21)	0.600	0.22 (0.05, 0.82)	0.024	0.58 (0.18, 1.79)	0.333
**Guardians' education**										
Primary school or below vs. college or above	2.35 (0.4, 14.93)	0.400	10.05 (1.35, 92.73)	0.013	3.09 (0.54, 19.23)	0.266	1.12 (0.16, 8.07)	0.908	0.50 (0.09, 2.55)	0.398
Middle school vs. college or above	1.21 (0.35, 4.2)	0.756	1.82 (0.51, 6.93)	0.341	1.19 (0.38, 3.84)	0.771	0.29 (0.07, 1.07)	0.096	0.28 (0.08, 0.92)	0.029
**Diagnosis**										
Acute lymphoblastic leukemia vs. others	0.87 (0.26, 2.9)	0.817	0.70 (0.19, 2.41)	0.567	0.83 (0.26, 2.62)	0.763	1.16 (0.33, 4.15)	0.831	0.58 (0.19, 1.78)	0.366
Non-Hodgkin's lymphoma vs. others	0.59 (0.15, 2.29)	0.445	0.53 (0.13, 2.13)	0.407	0.67 (0.18, 2.41)	0.564	1.39 (0.34, 5.9)	0.670	0.99 (0.29, 3.43)	0.990
Disease duration (months)	1 (0.95, 1.03)	0.695	0.97 (0.92, 1.01)	0.105	0.99 (0.95, 1.02)	0.537	0.95 (0.89, 1)	0.192	0.99 (0.96, 1.02)	0.717
ECOG (poor vs. good)	5.88 (2.08, 20.03)	0.003	9.09 (2.56, 50.04)	< 0.001	3.70 (1.35, 11.10)	0.018	12.50 (3.85, 47.62)	< 0.001	2.94 (1.11, 9.09)	0.043

## Discussion

Health-related quality of life of pediatric cancer patients is of great importance in optimizing oncological care for children. With the prolonged survival time and the consequently growing number of childhood cancer survivors, evaluating the HRQoL of such populations became both a medical and public health necessity. This study selected a sample of children with HM and evaluated their HRQoL with an age-appropriate generic instrument EQ-5D-Y. We found that the health utility of HM children was 0.88 on average and the self-rated global health VAS score had a mean of 85.8. The clinical index ECOG had strong correlations with the overall HRQoL indices and all the five EQ-5D-Y domains. Family income, disease duration, guardian's education, and residence were also significant factors exerting a differential effect on utility, VAS, “looking after myself,” and specific domains.

It is well-known that patients with cancer had worse health status than their healthy peers. In our study, the global health rated as EQ-5D-Y VAS was 85.8 on average. Compared to the healthy school-going children in China who achieved a mean VAS of 87.6 (16), the difference seems to be negligible. However, the HRQoL profile of our sample did not appear as satisfactory as those of healthy populations. There were 33.3–41.7 and 1.0–7.3% of our sample reporting “some problems” and “a lot of problems,” respectively on “mobility,” “looking after myself,” and “doing usual activities” dimensions. These numbers were much higher than the proportions (2.4–7.8 and 0.1–1.9%, respectively) reported in the general Chinese population (20). The findings indicate that HMs may have negative impacts on the physical functions of patients. Similar results have been reported in another study in mainland China showing that children with HMs have a lower level of physical activity (21). The dimension “looking after myself” presented the highest prevalence of problems among the five health dimensions. This can be explained by the fact that children/adolescents were not mature enough to take care of themselves, let alone those inflicted with a malignancy. The two psychological-related dimensions, “having pain or discomfort” and “feeling worried, sad or unhappy” also captured 38.5 and 42.7% of patients reporting problems, which were higher than the proportions (17.6 and 20.5%) reported in the general students (20). It has been acknowledged that treatments such as radiotherapy and chemotherapy could lead to fatigue and uncomfortableness (21), thus the patients tend to be stressed, depressed, and anxious. The relationship between the patients with HM and mental health was also identified previously (22).

The proxy rating has an important role in assessing the HRQoL of pediatric patients. ECOG grades the physical function of a patient with cancer by the doctor-in-charge. Our study showed that ECOG is the strongest factor predicting the concurrent utility, VAS, and all five EQ-5D-Y dimensions. Patients who were graded poor health in ECOG performance scores had lower utility and EQ-VAS scores. A similar relationship has been observed in Chinese adult patients with HM (23). As for the individual domains, more problems were reported if the patients were graded as having poor ECOG scores with an effect size (OR) ranging from 2.94 on “Feeling worried, sad or unhappy” to 12.50 “have pain or discomfort.”

Standing on the view of HRQoL measurement, health status evaluated with the ECOG system and HRQoL instrument are concordant for our sample of pediatric patients with HM. Both ECOG and HRQoL are independent health evaluations performed by clinicians and patients themselves. It has been suggested that the health status rated by the pediatric patients alone is not comprehensive (24) where bigger differences existed between proxy-reported and self-reported health outcomes in measuring pediatric patients than adults (25). Therefore, the agreement between the two health indicators can serve as a validation of our results. Considering ECOG is a well-established clinical approach assessing the health outcome of patients with cancer (26), ECOG and HRQoL measurements are complementary to each other to better understand patients' health and clinical needs.

The educational level of guardians was also found to have significant associations with dimensions of “looking after myself” and “feeling worried, sad or unhappy.” The findings were consistent with a study conducted among children with lymphoblastic leukemia in mainland China (22). Additionally, patients in rural areas had fewer problems with “looking after myself” and “Doing usual activities” dimensions compared to those living in urban areas, this might be due to the fact that patients residing in urban areas would rely more on caregivers and be less independent than children in the rural places.

Nowadays, pediatric patients with HM can survive to adulthood with a generally normal life expectancy. The care after the acute treatment phase bears great significance for this patient population to live a life as good as their peers, which is achievable as they are in the development and maturity processes. Targeted recovery care, home care, and patient and guardian's education should be promoted to improve the HRQoL of pediatric patients considering their particular physical, psychological, and clinical conditions. The effect of such interventions has been reported in interventional studies (27–30).

EQ-5D-Y is new to Chinese clinicians yet its wide use in clinical practice is expected in the near future, seeing that validation studies are either ongoing or published in Chinese children (3, 16). Pioneering studies such as the present one can shed a light on its performance in the target population. The ceiling effect of EQ-5D-Y was not prominent in this study, as only 21.88 and 18.75% of the sample reported full health in utility and VAS. A higher ceiling effect of 44.1% was observed in measuring healthy children (16). The above phenomenon is in accordance with clinical reality. It further shows that HRQoL assessed by EQ-5D-Y in pediatric patients with HM is unlikely to be biased upward by the ceiling effect. The correlation of VAS and utility in our study was moderate at a coefficient of 0.51, whereas in the adult population, these two EQ-5D measures are always strongly correlated. This may reflect an issue of separation between VAS and utility in measuring HRQoL of pediatric patients. The VAS was given by the children themselves to show their personal perception of health, whereas utility was calculated against the value set usually developed on the preference of adults, that is, taxpayers according to the principle (31). Controversy has not been solved about whose preference should be used to establish the value set for the health status of children (24). The current study was obliged to apply the Japanese value set reflecting the preference of adults aged 20–79 years (17). However, a child tends to be more optimistic about their status than adult proxies (32). The separation between VAS and utility may limit the usefulness of potential cost-utility analysis on pediatric patients. The optimal services recommended by a cost-utility analysis may not serve the target population as well as it is projected. Additionally, our findings direct to the need of conducting research on China-specific value sets.

Currently, studies investigating the health utility of pediatric hematological malignancies are rare in China. Consequently, economic evaluations based on utility will lack an important input of health outcomes to inform policymaking in pediatric cancer. There is a real need for health utility studies given the huge health burden caused by the HM. The present study may have made some contributions to that.

Our study is with several limitations. The sample comprised 96 inpatients only, which might be too small and too selective to represent the entire population of interest. The utility is likely to be underestimated as outpatients are generally better off than inpatients. The Japanese value set may not be fully transferrable to Chinese patients, which may introduce certain imprecisions in the utility estimates. Due to the limitations of data accessibility, patients' treatment information is lacking. The impact of medical interventions on the HRQoL cannot be explored in the study. Finally, the cross-sectional nature has precluded an effective inference on the casual effect relationship.

## Conclusion

Chinese pediatric patients with HM live in relatively poor health status and their HRQoL is of great concern to healthcare. The strong associations of health utility, VAS, and individual health dimensions with the traditional clinical index ECOG show that the evidence of ECOG and EQ-5D-Y are complementary to each other for producing a holistic evaluation of a patient's health. Therefore, the EQ-5D-Y makes a valuable tool for clinical decision-making and further generates utility to inform resource allocation in the oncological care of pediatric HM.

## Data availability statement

The raw data supporting the conclusions of this article will be made available by the authors, without undue reservation.

## Ethics statement

The studies involving human participants were reviewed and approved by Institutional Review Board of School of Public Health, Fudan University. Written informed consent to participate in this study was provided by the participants' legal guardian/next of kin.

## Author contributions

YS and H-JZ conceptualized the study, analyzed the data, and drafted the manuscript. AS interviewed the patients and managed the database. BW and WW handled the issues of project operation, data quality, and interpreted the results. NL designed the study and optimized the questionnaire. PW conceptualized the study, oversighted the project, and wrote several sections. All authors revised the manuscript critically and approved the manuscript for publication.
